# The Immunogenicity of Colorectal Cancer in Relation to Tumor Development and Treatment

**DOI:** 10.3390/ijms17071030

**Published:** 2016-06-29

**Authors:** Natasja L. de Vries, Marloes Swets, Alexander L. Vahrmeijer, Marianne Hokland, Peter J. K. Kuppen

**Affiliations:** 1 Department of Surgery, Leiden University Medical Center, Albinusdreef 2, P.O. Box 9600, 2300 RC Leiden, The Netherlands; N.L.de_Vries@lumc.nl (N.L.d.V.); M.Swets@lumc.nl (M.S.); A.L.Vahrmeijer@lumc.nl (A.L.V.); 2 Department of Biomedicine, Aarhus University, Bartholins Allé 6, Build. 1242, DK-8000 Aarhus, Denmark; mhokland@biomed.au.dk

**Keywords:** colorectal cancer, immune system, cancer immune interactions, cancer immunoediting, cancer treatment, tumor immunogenicity

## Abstract

Although most cancer types have been viewed as immunologically silent until recently, it has become increasingly clear that the immune system plays key roles in the course of tumor development. Remarkable progress towards understanding cancer immunogenicity and tumor-immune system interactions has revealed important implications for the design of novel immune-based therapies. Natural immune responses, but also therapeutic interventions, can modulate the tumor phenotype due to selective outgrowth of resistant subtypes. This is the result of heterogeneity of tumors, with genetic instability as a driving force, and obviously changes the immunogenicity of tumors. In this review, we discuss the immunogenicity of colorectal cancer (CRC) in relation to tumor development and treatment. As most tumors, CRC activates the immune system in various ways, and is also capable of escaping recognition and elimination by the immune system. Tumor-immune system interactions underlie the balance between immune control and immune escape, and may differ in primary tumors, in the circulation, and in liver metastases of CRC. Since CRC immunogenicity varies between tumors and individuals, novel immune-based therapeutic strategies should not only anticipate the molecular profile, but also the immunological profile of a specific tumor.

## 1. Introduction

Traditionally, cancer was proposed to be a cell-autonomous disease involving dynamic alterations in the genome [1]. New observations have led to a refinement and extension of the original biology of cancer, with a major focus on the tumor microenvironment, including the immune system [2]. Although the concept of immunological surveillance of cancer was already proposed more than 50 years ago [3], the role of the immune system in cancer development is yet to be fully elucidated. During the last decade, it has become increasingly evident that the immune system plays an important role in the course of tumor growth. As a consequence, novel immune-based therapeutic strategies are becoming available for the treatment of different types of cancer.

Colorectal cancer (CRC) is the third most frequent cancer type worldwide [4]. Approximately one million new cases of CRC per year arise throughout the world, with more than half a million deaths annually. For colorectal tumors that have not spread to distant sites, surgery is the primary treatment. However, in around 25% of CRC patients the disease has already metastasized at the time of diagnosis, and a further 30%–50% of patients diagnosed with early stage CRC develop metastatic disease during clinical follow-up [5]. One of the major challenges that need further research is the fact that curative treatment options are limited for these patients. Novel immune-based therapeutic strategies are becoming available, which may be highly valuable for the treatment of CRC patients [6].

Until recently, CRC was considered to be immunologically silent, possessing limited ability to provoke an immune response. Remarkable progress in cancer immunology has served to change our view on CRC immunogenicity. At present, it is clear that both genetic and epigenetic alterations underlying the development of CRC contribute to the formation of immunogenic tumor-specific and tumor-associated antigens. These tumor antigens allow the identification and elimination of CRC cells by the immune system [7]. Importantly, heterogeneity of tumors, with genetic instability as a driving force, allows the outgrowth of immune escape variants [8]. Therefore, for the design of efficient immunotherapeutic strategies, it is crucial to understand the complex field of tumor-immune system interactions underlying the balance in tumor development between immune control and immune escape in CRC.

This review is aimed at discussing CRC immunogenicity in relation to tumor development, including tumor metastasis, and consequences for the design of novel immune-based therapies. We first discuss immunogenicity of CRC in connection with the genetic and epigenetic makeup of CRC. Next, the role of the immune system in CRC development is discussed with a particular focus on the distinct roles of tumor-immune system interactions in primary tumors, in the circulation, and in liver metastases. Finally, the importance of CRC immunogenicity in the design of immune-based therapeutic strategies for patients with CRC is discussed.

## 2. Genetic and Epigenetic Makeup of Colorectal Cancer (CRC)

**Figure 1:**
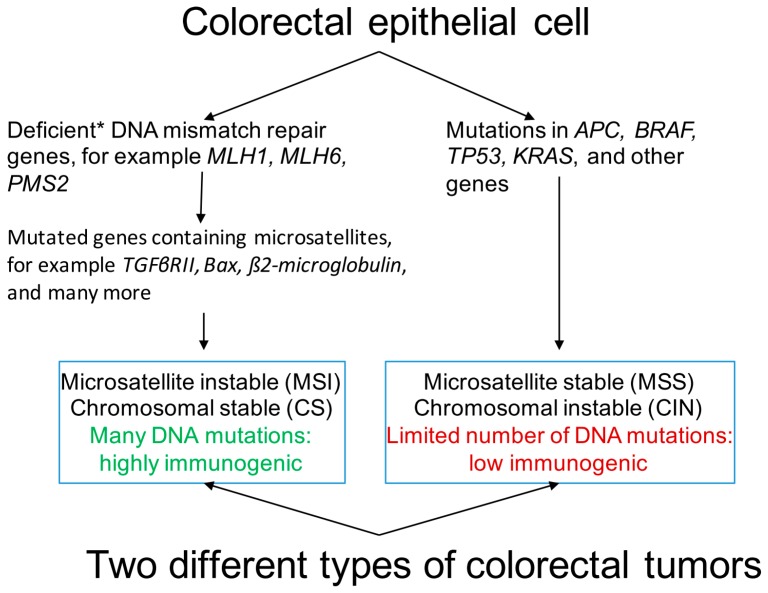
Colorectal cancer: two different types of tumors. Characteristics of the two main phenotypes of colorectal cancer are shown. * Deficiencies in DNA mismatch repair genes are due to genetic (DNA mutations) or epigenetic (methylation) aberrations in the genes coding for the responsible enzymes. The phenotype showing aberrant methylation is often indicated as the CpG island methylator phenotype (CIMP).

The development of CRC is a multi-stage process characterized by the accumulation of mutations in particular genes as it progresses through the classical adenoma-carcinoma sequence [9]. In CRC, at least three distinct pathways of genomic instability have been described: the chromosomal instability (CIN) or microsatellite stable (MSS) pathway, the microsatellite instability (MSI) pathway, and the CpG island methylator phenotype (CIMP) pathway (Figure 1) [10]. The majority of CRC arises through the adenoma-carcinoma sequence pathway, which is mainly governed by CIN. The CIN or MSS pathways are characterized by the accumulation of a characteristic set of mutations in specific oncogenes (*BRAF*, *KRAS*, *PI3K*) and tumor-suppressor genes (*APC*, *TP53*), leading to numerical and structural chromosomal aberrations and a high frequency of loss of heterozygosity [11]. Loss of function of *APC* is among the earliest biological alterations in CRC pathogenesis [12]. Accordingly, patients with germ line mutations in the *APC* gene, known as familial adenomatous polyposis, show numerous adenomatous polyps in the colon and rectum [13].

Another form of genomic instability involved in pathogenesis of CRC is MSI. Due to a deficient DNA mismatch repair (MMR) system, involving enzymes as MLH1, MLH6 and PMS2, numerous mutations arise at repetitive microsatellite DNA sequences [14] and affects genes that contain such microsatellites as *Bax*, *TGFβRII*, *β2-microglobulin* (Figure 1). This MMR-deficient phenotype is termed MSI, and is observed in 10%−20% of sporadic CRC cases [15]. In addition, around 3% of all CRC cases are associated with Lynch syndrome, also called hereditary non-polyposis CRC, caused by inactivating germ line mutations in MMR genes [16]. The MSI phenotype is characterized by right-sided location in the colon [17], and presence of numerous tumor-infiltrating lymphocytes (TILs) [18].

Finally, the CIMP pathway of pathogenesis has been described in CRC. CIMP is characterized by hypermethylation of promoter CpG island sites, which can result in repression of transcription of several tumor-suppressor genes [19]. Most cases of sporadic MSI CRC are CIMP positive, and commonly located in the proximal site of the colon [10]. Occasionally, tumors from CRC patients can exhibit features of multiple pathways of genomic instability [11].

As a result of the accumulation of mutations by the different pathways of genomic instability in CRC, proteins with a different amino acid sequence can arise. These aberrant proteins may lead to function loss, as in the case of the MMR proteins, but can also affect expression of other proteins, for instance when they involve key proteins in signaling pathways. Combined this leads to changes in protein expression, including over- and under-expression, in a much broader context than simply the mutated protein itself. In addition, almost all CRCs have epigenetic changes, such as aberrant DNA methylation that can, for instance, silence transcription of tumor-suppressor genes [20]. Thus, because of both genetic and epigenetic alterations, tumors from CRC patients express a range of aberrantly expressed proteins, which may represent targets for the immune system.

## 3. Immunogenicity of CRC

Immunogenicity of CRC, meaning its ability to induce an immune response, has long been debated. Traditionally, immune responses were not considered to play a significant role in CRC growth. However, it is becoming increasingly clear that CRC cells are not immunologically silent. New observations have shown that the immune system plays an important role in the course of CRC development [21]. For a successful adaptive immune response, CRC cells have to possess immunogenic antigens (“signal 1”) that can be recognized during the process of immune surveillance. Hence, colorectal tumor antigens must be presented to the immune system for anti-tumor immune responses to occur. This generally requires the presentation of tumor antigens to T cells by the major histocompatibility complex (MHC) either directly by the tumor cell, or via interactions with dendritic cells (DCs), attracted and activated due to tissue damage caused by the growing tumor (“signal 0”), that can cross-present exogenous tumor-derived antigens to T cells. Co-stimulatory signals (“signal 2”), provided by, among others, the tumor cells themselves, T helper cells, and DCs may further fuel cytotoxic T cell responses. Recently, a novel class of tumor-specific antigens derived from mutated proteins, only present in tumor cells and called tumor neo-antigens, has been described [22]. Tumor neo-antigens are absent in the thymus and are thus not affected by central T cell tolerance that would normally ensure the deletion of high-affinity T cells by negative selection [22]. Interestingly, anti-tumor immune responses were found to positively correlate with expression of tumor neo-antigens on the tumor cell surface in CRC [7].

As noted above, aberrantly expressed proteins in CRC, through mutations or overexpression, represent potential immunogenic targets. Tumor-associated antigens commonly expressed in CRC include mucin 1 (MUC1), carcinoembryonic antigen (CEA), human epidermal growth factor receptor 2 (HER2)/neu, epidermal growth factor receptor (EGFR), epithelial cell adhesion molecule (EpCAM), telomerase, heparanase, and tumor protein p53 (TP53). Emerging evidence shows that such tumor (neo-)antigens induce tumor-specific T cell reactivity [22]. In approximately 50% of CRC, mutations in tumor-suppressor gene *TP53* are found, frequently resulting in overexpression of mutant TP53 protein in tumor cells [23]. As a consequence, *TP53* mutations may lead to enhanced processing and presentation of immunogenic antigens by tumor cells for recognition by the immune system. It has been demonstrated that both cellular as well as antibody-mediated immune responses against TP53 can be detected in CRC patients [24,25]. This has prompted the use of TP53 as a tumor-associated antigen for the design of novel immune-based therapeutic strategies in CRC. Clinical trials aiming at therapeutic vaccination with wild-type human TP53 have been performed in CRC patients, which showed the feasibility of inducing TP53-specific T cell reactivity upon vaccination [25,26]. These findings indicate a role for tumor antigen-specific T cell reactivity in CRC development.

The distinct pathogenic pathways of CRC are correlated with different levels of immunogenicity. CRC caused by MSI, generally chromosomal-stable and CIMP-positive, have been proposed to show enhanced immunogenicity compared to CRC classified as MMR proficient or MSS, which mostly show CIN [27,28] (Figure 1). It was demonstrated that the lymphocyte response to proteins extracted from MSS CRC was poor, whereas marked proliferation of lymphocytes was observed after challenging with proteins extracted from MSI CRC [28]. Among MSS CRC, CIN phenotype was reported to be more immunogenic than chromosomal stable tumors, as indicated by a very low lymphocyte response to chromosomal stable tumor proteins [28]. Interestingly, a prominent presence of TILs has been found in association with MSI CRC, with a predominant cytotoxic phenotype (CD8^+^) [18]. Furthermore, studies have indicated that patients with MSI CRC showed reduced metastasis rate and survival benefit compared to patients with MSS CRC [29]. It was proposed that these differences between MSI and MSS CRC are the result of the high mutational load of CRC exhibiting the MSI phenotype [28]. This high mutational load may generate high antigenicity with an associated up-regulation of immunogenic antigens that could be recognized by the immune system [28,30]. However, CRC exhibiting the MSI phenotype was also found to be frequently deficient for MHC class I molecules on their cell surface [31], and thus expected to evade recognition and elimination by T cells. In contrast, loss of MHC class I was less frequent in MSS CRC [31]. As such, the presence and function of tumor-specific T cells in MSI CRC and the prognostic value thereof remains to be explored further.

## 4. Tumor-Immune System Interactions in CRC

The genetically unstable nature of CRC may result in the onset of tumor cell variants that are capable of escaping recognition and elimination by the immune system [8]. Natural immune responses, but also therapeutic interventions, can modulate the tumor phenotype due to selective outgrowth of resistant subtypes. For the design of efficient immune-based therapies for CRC, it is crucial to understand and predict tumor-immune system interactions underlying the balance in tumor development between immune control and immune escape. Multifaceted interactions between colorectal tumor cells and immune cells take place within primary tumors, but also in the circulation and in liver metastases. The distinct features of these tumor-immune system interactions will be discussed below.

### 4.1. Tumor-Immune System Interactions in Primary Tumors

The role of the immune system in primary colorectal tumors has been extensively studied. An early event in tumor growth is activation of the immune system, resulting from chemokines and cytokines that are released due to tissue damage caused by the growing tumor [32]. Multiple studies have revealed that primary tumors are, in general, densely infiltrated by different T cell subsets [33,34,35,36]. It was found that presence of a dense infiltration of CD8^+^ T cells correlated with a reduced risk of tumor recurrence and improved patient survival compared to low infiltration of CD8^+^ T cells [34,36,37]. In particular, presence of a high density of cytotoxic memory T cells in primary tumors, compared to low density, was correlated with a reduced risk of recurrence and metastasis, and an improved survival for CRC patients [33,35,38]. Importantly, tumor-infiltrating CD8^+^ T cells showed reactivity against tumor antigens in some CRC patients [39,40]. CD8^+^ T cells responding to CRC-associated antigen MUC1 were observed [39], as well as T cells responding to CRC-associated antigens HER2/neu, CEA, and TP53 [40]. It was demonstrated that primary tumors showed enhanced tumor-selective activity and cytotoxicity of CD8^+^ T cells compared to normal mucosa in CRC patients [39]. This tumor-selective activation and cytotoxic activity was found in subpopulations of CD8^+^ T cells in CRC patients [39,40]. Hence, the prognostic relevance of TILs in CRC may be restricted to subpopulations of tumor antigen-specific CD8^+^ T cells. As a consequence, the number of tumor antigen-specific CD8^+^ T cells in primary tumors may be a better prognostic factor than CD8^+^ T cell infiltration in general [40].

Anti-tumor T cell responses are controlled by regulatory T cells (Tregs), which function as immunosuppressive cells to prevent excessive immune responses and to maintain immune tolerance [41]. Presence of a high density of tumor-infiltrating Tregs has been found in primary tumors [42,43]. This increased presence of tumor-infiltrating Tregs is believed to support tumor progression by mediating tumor-associated immunosuppression [41]. Contradictory results have been found for the prognostic significance of a high density of tumor-infiltrating Tregs in primary tumors. Some studies have shown that increased presence of tumor-infiltrating Tregs is correlated with a favorable prognosis [43], while others claim that it is correlated with disease progression in CRC [42]. One study found that Tregs showed reactivity against tumor antigens in peripheral blood samples of CRC patients [44], but no studies have focused on tumor antigen-specific Tregs in primary tumors as of yet.

Aberrant MHC class I expression, expected to result from escape from CD8^+^ T cell reactivity, is quite common in primary tumors, especially for MSI CRC [45,46]. Such tumor cells represent potential targets for natural killer (NK) cells, which are known to be able to recognize and kill cells with low or absent MHC class I expression [47]. A limited number of studies have focused on the contribution of NK cells to immune surveillance in CRC [48]. NK cells can be divided into two major subpopulations: CD56^dim^ and CD56^bright^ NK cells. The CD56^dim^ NK cell subset possesses the cytotoxic potential, whereas the CD56^bright^ NK cell subset is mainly involved in cytokine production [47]. Recently, Halama et al. performed a detailed quantification of infiltrating NK cells and T cells within normal tissues, adenomas, and primary tumors and liver metastases of CRC [48]. They demonstrated that NK cells were found in very low numbers within colorectal tumor tissue compared to adjacent normal mucosa, independently of MHC class I expression [48].

NK cell anti-tumor activity in primary tumors can be further impeded by expression of non-classical MHC class I molecules such as human leukocyte antigen (HLA)-E and HLA-G on CRC cells [49]. Both HLA-E and HLA-G can bind to NK cell inhibitory receptors, thereby inhibiting the cytotoxic activity of NK cells [50,51]. In addition, HLA-G can inhibit various other immune cell types such as T cells, DCs, and macrophages [52]. HLA-E has been found to be frequently expressed in primary tumors [53]; however, the prognostic significance is debated. Studies have shown that high levels of HLA-E are correlated with a poor prognosis in CRC [54], while other studies indicated an association between HLA-E expression and better clinical outcome in CRC [55]. In addition, primary tumors demonstrated expression of HLA-G [56], which generally correlated with a worse survival rate for CRC patients compared to HLA-G negative tumors [54].

A unique subset of T cells that express a T cell receptor complex in combination with various NK cell markers constitute natural killer T (NKT) cells [57]. Although their exact function is not fully determined as of yet, NKT cells are also involved in tumor immune surveillance and anti-tumor immune responses. It is hypothesized that NKT cells can rapidly produce pro-inflammatory cytokines upon stimulation, which can subsequently determine antigen-specific T cell responses [58]. The role of NKT cells in CRC has not yet been studied extensively. In primary tumors, increased infiltration of NKT cells has been observed, which correlated with a favorable prognosis for CRC patients [59]. Further studies are required to better determine their role in anti-tumor immune activity.

Whereas CD8^+^ T cells and NK cells are potent effector immune cells, immunosuppressive cells such as myeloid-derived suppressor cell (MDSC) and tumor-associated macrophage (TAM) subsets also play important roles in the tumor microenvironment [60,61]. MDSCs are a collection of immune cells that originate from the myeloid lineage, including myeloid progenitor cells, immature DCs, macrophages, and granulocytes [61]. MDSCs localize within the tumor microenvironment, where they regulate immune responses by interacting with other immune cells such as CD8^+^ T cells and NK cells, and suppress their cytotoxic functions [62]. Elevated levels of MDSCs have been observed in primary CRC [63,64], but prognostic significance of elevated levels of MDSCs for clinical outcome of CRC patients has not been studied as of yet.

TAMs are derived from circulating monocyte precursors, including MDSCs, and are located in the tumor microenvironment. Controversial roles for TAMs have been described: they can either promote or inhibit tumor progression and formation of metastases [60]. These different functions of TAMs are sometimes referred to as M1 macrophages (anti-tumorigenic) or M2 macrophages (pro-tumorigenic), although their polarization status is much more complex due to a high degree of plasticity [65]. Few studies have demonstrated that presence of TAMs and their subtype distribution in primary tumors are associated with clinical outcome of CRC patients [66,67,68]. It was found that both TAM subsets, M1 and M2, were found to be present at increased levels in primary tumors [68]. Presence of M1 macrophages has been correlated with better clinical outcome [66], whereas expression of M2 macrophage markers has been correlated with worse clinical outcome in CRC [67].

**Figure 2:**
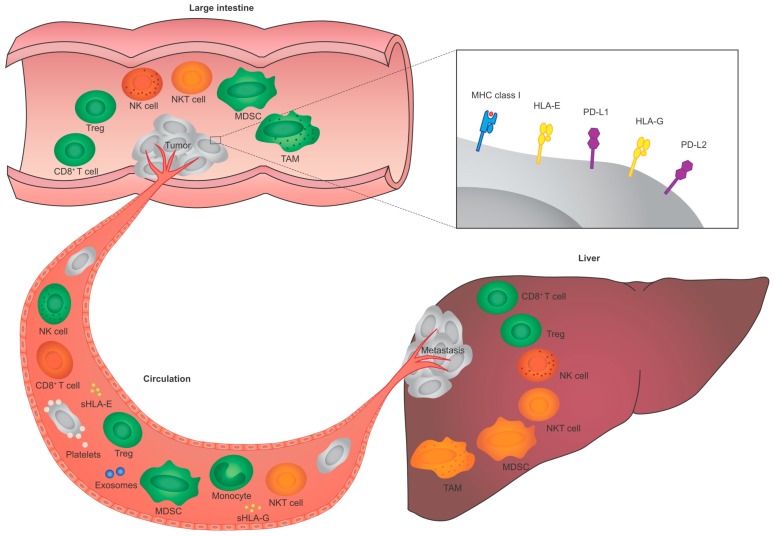
Schematic representation of tumor-immune system interactions in the primary tumor, in the circulation, and in liver metastasis of CRC. Interactions between various types of immune cells and tumor cells take place in CRC, which are immunologically different in primary tumors (**upper** part), in the circulation (**left** part), and in liver metastases (**right** part). In each of these compartments, immune cell types can either affect tumor development (colored in green), have no effect on tumor development (colored in red), or their effect on tumor development is not yet clear in CRC (colored in orange). Abbreviations: CRC, colorectal cancer; HLA, human leukocyte antigen; MDSC, myeloid-derived suppressor cell; MHC, major histocompatibility complex; NK, natural killer; NKT, natural killer T; PD-L1, programmed death-ligand 1; PD-L2, programmed death-ligand 2; sHLA, soluble HLA; TAM, tumor-associated macrophage; Treg, regulatory T cell.

Taken together, tumor-immune system interactions play an important role in primary CRC (Figure 2). T cells are found in high numbers in primary tumors, whereas NK cells are generally scarce. Tumor-specific T cells reactive with various CRC-associated antigens are present in primary CRC. Hence, primary CRC is immunogenic and induces immune responses. Modulation of key proteins in tumor-specific T cell responses may be an important immune escape strategy for primary CRC cells.

### 4.2. Tumor-Immune System Interactions in the Circulation

Although many studies have focused on the role of the immune system in primary colorectal tumors, little is known about the role of immune cells in peripheral blood and the lymphatic system in tumor progression and metastasis. During the process of dissemination, CRC cells detach from the primary tumor and intravasate into the circulation, resulting in a different immune interaction. In primary tumors, immune interactions are dependent on signals such as chemokines and cytokines that cause immune cell infiltration in the tumor. In contrast, in the peripheral blood and lymphatic system, tumor cells may randomly contact immune cells. The entering of tumor cells into the peripheral circulation is accompanied by epithelial-mesenchymal transition (EMT) [69]. In EMT, carcinoma cells in primary tumors lose their cell-cell adhesions, and acquire the motility and invasive features to become mesenchymal cells [69]. The acquirement of an EMT-like phenotype with enhanced metastatic potential was found to be modified by presence of platelets in the vasculature [70]. Recently, it has been demonstrated that aspirin use in CRC patients was associated with favorable clinical outcome if primary tumors expressed MHC class I [71]. It was suggested that this was the result of aspirin-mediated inhibition of tumor-platelet signaling [71]. Whether circulating CRC cells indeed retain the same expression of MHC class I as the primary tumor remains to be elucidated.

In addition to platelet-tumor interactions, CRC cells encounter different types of immune cells in the circulation. Interestingly, it was shown that CRC patients with down-regulation or loss of MHC class I expression in their primary tumors developed fewer distant metastases [45]. This finding supports the hypothesis that NK cells in the circulation play an active role in eliminating disseminated tumor cells in CRC patients. NK cells form 10%−15% of the peripheral blood lymphocyte population [72]. Approximately 90% of peripheral blood NK cells belongs to the CD56^dim^ NK cell subset, while the remaining 10% consists of CD56^bright^ NK cells [72]. Recent studies have demonstrated that peripheral blood NK cells from CRC patients have a deregulated phenotype mainly characterized by reduced expression of activating (natural cytotoxicity) receptors [73,74]. These findings suggest that CRC patients show an impairment of NK cell activity in peripheral blood, which may facilitate the dissemination of tumor cells. NK cell activity in peripheral blood may for instance be impaired by the presence of soluble variants of HLA-E (sHLA-E) and HLA-G (sHLA-G) in the circulation of CRC patients as a result of shedding of the membrane-bound protein [75,76]. Increased levels of sHLA-E and sHLA-G in the blood circulation have been reported in patients with CRC compared to healthy donors [77,78]. It was suggested that, similar to cell surface expression of HLA-E and HLA-G, both sHLA-E and sHLA-G inhibit the cytotoxic activity of NK cells in the circulation [79,80]. In addition to sHLA-E and sHLA-G, other soluble factors are present in the circulation that are involved in anti-tumor immune responses, including exosomes [81]. Exosomes are small membrane vesicles released by cells into the extracellular environment, which can contain proteins, lipids, mRNAs, and miRNAs, depending on the origin of the secreting cells [81]. Recently, CRC-derived exosomes have been found to play a role in promoting tumor growth and stimulating angiogenesis, and the level of circulating exosomal miRNA was found to be a biomarker for CRC recurrence [81,82].

Several studies showed the presence of tumor-specific T cells in peripheral blood of CRC patients, comprising CD8^+^ T cells reactive with CRC-associated antigens including TP53, MUC1, EpCAM, HER2/neu, CEA, and heparanase [39,44,83]. However, the presence of tumor-specific CD8^+^ T cells in peripheral blood was not correlated with improved clinical outcome of CRC patients [83].

In peripheral blood of CRC patients, as compared to healthy donors, increased levels of immunosuppressive Tregs have been observed [84,85]. It was shown that these increased levels of Tregs correlated with tumor stage [84,85]. Importantly, tumor antigen-specific Tregs have been found in peripheral blood of CRC patients for a limited repertoire of tumor antigens [44]. Presence of Tregs specific for CRC-associated antigens CEA, telomerase, HER2/neu, and MUC-1 were most prevalently detected in a part of the CRC patients [44]. This suggests that peripheral blood Tregs can mediate T cell suppression in an antigen-selective way.

As is the case in primary tumors, CRC patients, as compared to healthy donors, showed increased numbers of immunosuppressive MDSCs in peripheral blood [63,86]. Furthermore, it was found that the number of MDSCs in peripheral blood of CRC patients correlated with tumor stage [63,86]. These findings suggest an important role of MDSCs in the inhibition of anti-tumor immune responses in peripheral blood of CRC patients. The role of peripheral blood monocytes in CRC patients has not yet been studied in detail. In general, a high frequency of circulating monocytes was found in CRC patients compared to healthy donors, which was associated with advanced tumor stages [68]. Finally, no studies on the subtype distribution of NKT cells in peripheral blood of CRC patients and clinical outcome have been performed as of yet. Hence, their role in anti-tumor immune responses in peripheral blood of CRC patients remains to be explored further.

In addition to the peripheral blood, tumor-immune system interactions in the lymphatic system also play important roles in CRC progression. Metastases usually show up first in draining lymph nodes, where they typically present tumor antigens to the immune system to initiate anti-tumor immune responses. In lymph node metastases from CRC, an increased presence of CD8^+^ T cells [87], Tregs [88], and NKT cells [59] has been found. Presence of a high density of these immune cell types, as compared to low density, was associated with improved prognosis [59,87,88]. Furthermore, it has been shown that NK cells are present in lymph nodes, but that they display a different phenotype than peripheral blood NK cells [89]. The anti-tumor activity of NK cells in lymph nodes of CRC patients and the prognostic value thereof remain to be elucidated.

Taken together, tumor-immune system interactions, characterized by direct contact, play an important role in the circulation (Figure 2), and are immunologically different from primary CRC. As compared to primary CRC, NK cells may play a more active role in preventing the dissemination of tumor cells in the circulation. These different tumor-immune system interactions in primary CRC and in the circulation obviously affect tumor immunogenicity. The phenotypic changes of tumor cells associated with the process of dissemination, as well as their immunogenicity, remains to be explored further.

### 4.3. Tumor-Immune System Interactions in Liver Metastases

Once primary CRC cells acquire the ability to evade immune surveillance in the primary tumor and in the circulation, they mainly metastasize to the liver. Little is known about the role of the immune system in liver metastases of CRC. Studies have shown the presence and prognostic value of TIL densities in liver metastases of CRC [90,91,92,93]. It was found that CRC patients showed presence of a high density of CD8^+^ T cells in liver metastases [90,91,92]. This presence of a high density of CD8^+^ T cells in liver metastases, as compared to low density, was correlated with a favorable clinical outcome in patients who underwent resection of colorectal liver metastases [91]. The prognostic value of a high density of T cells in resected colorectal liver metastases was found to be improved by expression of MHC class I on tumor cells [93]. The localization and distribution of T cells was mainly observed at the invasive margin of liver metastases [92]. It was demonstrated that liver metastases of CRC patients showed enhanced tumor-selective activation and cytotoxic activity of CD8^+^ T cells compared to the surrounding normal liver tissue [90]. Importantly, CD8^+^ T cells infiltrating liver metastases showed reactivity against tumor antigens for some of the CRC patients [40,90]. CD8^+^ T cells responding to CRC-associated antigens MUC1, HER2/neu, CEA, TP53, and heparanase were observed [40,90], without apparent differences as compared to primary tumors [40]. However, only a small minority of CD8^+^ T cells in liver metastases showed reactivity against tumor antigens [90]. Hence, CD8^+^ T cells are present in liver metastases of CRC patients, but their tumor-specific cytotoxic activity may be dependent on the presence of tumor antigens, MHC molecules, and immunosuppressive cells.

Although immunosupressive Tregs are generally scarce in liver metastases, the presence of high numbers of Tregs relative to CD8^+^ or CD4^+^ T cells within liver metastases was associated with an unfavorable outcome in patients who underwent resection of colorectal liver metastases, as compared to presence of relative low numbers of Tregs [94]. The reactivity of Tregs against tumor antigens in liver metastases of CRC patients has not been studied as of yet.

Few studies have investigated the expression of MHC in primary tumors and associated liver metastases [95,96]. One study showed that HLA class I, as well as TP53, was concordantly expressed in the majority of primary tumors and their associated liver metastases [95]. A recent study confirmed the concordance between the expression level of MHC class I in primary tumors and their associated liver metastases, but showed that the majority of HLA-G-positive tumors did not express HLA-G in their associated liver metastases [96]. Similarity between the expression of HLA-E in primary tumors and associated liver metastases is still unclear. Further studies are required to determine the exact contribution of the expression of the different MHC molecules on tumor cells to formation of metastases in CRC.

Besides T cells, the liver contains a large population of NK cells accounting for up to 30% of human hepatic lymphocytes [97]. This is in contrast with the lower frequency of NK cells in blood (10%–15%) or other peripheral tissues such as lymph nodes. Despite the high presence of NK cells in the liver, they have been shown to poorly infiltrate liver metastases compared to other lymphocytes such as T cells [98,99]. Interestingly, hepatic NK cells have been found to be functionally distinct from peripheral blood NK cells, mainly characterized by a more immune tolerogenic phenotype [100]. The percentage of CD56^dim^ NK cells decreased from 90% of peripheral blood NK cells to approximately 50% of hepatic NK cells, with a corresponding increased percentage of CD56^bright^ NK cells from 10% of peripheral blood NK cells to 50% of hepatic NK cells [101]. These findings suggest that hepatic NK cells are equally involved in cytotoxic function and immune regulation.

The liver is also enriched in NKT cells, accounting for up to 25% of human hepatic lymphocytes [97]. The role of NKT cells in liver metastases of CRC patients is not determined as of yet. One study showed that, despite the relative abundance of NKT cells in the liver, their presence was found to be decreased in liver metastases as compared to non-metastatic livers [98]. The effect of decreased presence of NK cells and NKT cells in liver metastases on CRC patients’ clinical outcome is unclear, and could be explored further.

Finally, little is known about the presence and prognostic value of immunosuppressive MDSCs and TAMs within liver metastases of CRC patients. An immunosuppressive state favors tumor progression and metastasis, in which MDSCs and TAMs obviously play important roles. Recently, a study demonstrated that progression of liver metastases was promoted via the recruitment of MDSCs [102]. Although TAMs are associated with tumor progression, including metastasis, in CRC, their role in liver metastases has not yet been determined. One study showed that the effects of TAMs on liver metastasis of CRC did not depend on the total number of TAMs, but on the number and proportion of M1 and M2 subsets, and that presence of M2 subset and high M2/M1 ratio were predictors for liver metastases [103]. Further studies elucidating a direct role for MDSCs and TAMs in anti-tumor immune responses in liver metastases of CRC patients are required.

Taken together, tumor-immune system interactions play an important role in liver metastases of CRC patients (Figure 2), and are immunologically different compared to those in primary tumors and the circulation. It is hypothesized that metastases may be less immunogenic as compared to primary tumors and circulating tumor cells, as they develop under active immune surveillance in the primary tumor and circulation [104]. However, initial studies indicate the expression of tumor antigens, MHC molecules, tumor-specific T cell responses, and the presence of immunosuppressive cells in colorectal liver metastases, principally not different from primary tumors. Hence, the immunogenicity of colorectal liver metastases remains to be further studied.

## 5. Role of Immune Checkpoints in CRC

Although the aforementioned findings indicate that tumor antigen-specific T cells are present in CRC, tumor-associated immunosuppression can impair their anti-tumor activity. As the presence of high numbers of tumor-specific T cells is correlated with a favorable prognosis in CRC patients [40], the rationale of overcoming tumor-associated immunosuppression in the tumor microenvironment has emerged as a novel target for anti-cancer treatment. Activation of T cells is dependent on the balance between co-stimulatory (such as OX40, CD27, or CD137) and co-inhibitory signals from cell surface receptors. These immune checkpoints are crucial for maintaining immune self-tolerance and limiting excessive tissue damage. Different inhibitory receptors are expressed on the cell surface of T cells, including cytotoxic T-lymphocyte-associated antigen 4 (CTLA-4) and programmed death 1 (PD-1), which prevent T cell activation by different mechanisms of action [105,106]. CTLA-4 decreases T-cell immune response by binding to B7 protein on activated antigen-presenting cells, thereby preventing the co-stimulation with CD28 [105]. PD-1 inhibits T cell activity by binding to two distinct members of the B7 family, namely programmed death-ligand 1 (PD-L1) and 2 (PD-L2) [106]. Up-regulation of expression of PD-1 ligands plays an important role in tumor-immune system interaction. Both PD-L1 and PD-L2 have been reported to be expressed by various different types of tumor cells, thereby providing inhibitory signals to suppress activity of T cells [107]. Thus, tumors can modulate the expression of immune checkpoint proteins as an important immune resistance mechanism. Monoclonal antibodies blocking these inhibitory immune checkpoints have recently been shown to be very successful for the treatment of melanoma [108] and non-small cell lung cancer [109].

The significance of immune checkpoint inhibition for treatment of CRC patients is currently being investigated, and the first successes have been reviewed by Singh et al. [110] and Jacobs et al. [111]. Interestingly, blockade of immune checkpoint inhibitors was found to be especially effective in metastatic CRC patients exhibiting the MSI phenotype as compared to the MSS phenotype [112]. It was also demonstrated that MSI CRC showed a more than 20-fold increase in the number of mutation-associated neo-antigens compared to MSS CRC [112]. As discussed previously, MSI CRC show dense immune infiltration in tumors compared to MSS CRC, which was directed against mutation-associated neo-antigens [30]. Thus, the driving force of immune checkpoint inhibition of T cells in MSI CRC is consistent with immunogenic neo-antigens recognized by tumor-specific T cells. Recently, a study indicated that the immune microenvironment of MSI CRC relative to MSS CRC highly up-regulated expression of several immune checkpoints, including PD-1 and CTLA-4 [113]. Furthermore, another recent study indicated that MSI CRC is more likely to show expression of PD-1 on lymphocytes and PD-L1 on tumor cells, as compared to MSS CRC [114]. These findings indicate that the active immune microenvironment in MSI CRC is counterbalanced by immune inhibitory signals resisting tumor elimination. Hence, blocking immune checkpoints can be especially effective in CRC exhibiting the MSI phenotype. Apparently, tumor-specific T cells are present, but functionally blocked. The combination of anti-PD-1 with anti-CTLA-4 monoclonal antibodies is currently evaluated in clinical trials for advanced CRC patients exhibiting the MSI phenotype [110].

## 6. Current Treatment Options and CRC Immunogenicity

The mainstay management of primary CRC is surgery, which can be combined with neoadjuvant or adjuvant chemotherapy or radiotherapy depending on tumor stage. Clinically, colon and rectal cancers are treated differently. For patients with colon cancer, the treatment standard is surgical resection, which can be followed by adjuvant chemotherapy depending on tumor stage [5]. For patients with rectal cancer, the procedure of choice is surgery alone, or short-course (chemo)radiotherapy before surgical resection, which can be followed by adjuvant chemotherapy depending on tumor stage [5]. These conventional therapies for CRC were originally developed based on the conception that cancer constitutes a cell-autonomous disease involving dynamic alterations in the genome and epigenome. Hence, chemotherapy and radiation have been employed as anti-cancer treatments based on their cytostatic and cytotoxic effects to ultimately kill tumor cells in situ by the accumulation of extensive DNA damage. However, increasing evidence suggests that the efficacy of chemotherapy and radiation may not only result from direct cytotoxicity, but also depend on tumor-specific immune responses [115,116].

During surgical resection, the colorectal tumor and its microenvironment, including tumor-specific T cells, are completely removed. In contrast, both chemotherapy and radiation induce cell death by the accumulation of extensive DNA damage. It has been shown that the resulting mass of dead cancer cells in situ is still capable of activating tumor-specific immune responses [117]. Due to their cytotoxic effects on tumor cells, chemotherapy and radiation can provide a source of degraded tumor antigens for cross-presentation by DCs, which in turn can induce tumor antigen-specific immune responses. Importantly, this mass of dead tumor cells in situ was found to provide at least similar antigenic diversity as the tumor itself [118]. Cell death induced by chemotherapy and radiation can thus trigger anti-tumor immunity that can facilitate tumor rejection. In case of loco-regional radiotherapy, the local mass of immunogenic dead cells has been shown to be able to support the destruction of (distant) metastases [119]. Hence, radiation may support not only local but also systemic tumor immunity. Together, chemotherapy and radiation may promote CRC immunogenicity, mostly by inducing tumor cell death and thereby increased efficiency of tumor antigen presentation. In this way, conventional chemotherapy and radiation can contribute to the induction of effective anti-tumor immune responses. Interestingly, neoadjuvant treatment with radiotherapy has been shown to be able to result in a complete clinical response in rectal cancer patients [120]. Several studies have proposed a “watch and wait” policy for rectal cancer patients when no residual rectal tumor can be found after neoadjuvant radiotherapy [121,122]. They indicated that rectal cancer patients with complete clinical response in the “watch and wait” strategy resulted in similar local and distant disease control compared to patients treated by rectal resection [121,122]. This complete clinical response upon neoadjuvant radiotherapy in rectal cancer patients may involve a role for the immune system.

For patients with metastatic CRC, curative treatment options are limited. These patients frequently present with metastases in the liver, but the lungs, peritoneum or distant lymph nodes can also be affected. In case of confined metastases to the liver, surgical resection of liver metastases offers the best possibility of curative treatment, which can be combined with perioperative or adjuvant chemotherapy [5]. However, in the majority of patients, surgical resection is not feasible due to the number, size, or location of liver metastases. For these patients, minimally invasive loco-regional approaches such as local ablative therapies and loco-regional chemotherapy might offer palliation or prolongation of their survival [123]. It has been shown that several loco-regional therapeutic approaches for the treatment of liver metastases can also affect anti-tumor immune responses beyond their direct cytotoxicity to tumor cells. In isolated hepatic perfusion (IHP), cytotoxic agents are administered directly in the hepatic artery by a complex surgical technique [124]. Interestingly, it was shown that IHP resulted in a profound increase in antibody levels directed against a variety of tumor-specific antigens in serum samples of CRC patients with liver metastases compared to surgical resection and other ablative therapies [125]. Other studies showed that radiofrequency thermal ablation and laser-induced thermotherapy, which can also be used for the treatment of liver metastases, induced activation and cytotoxic activity of tumor-specific T cells in liver metastases [126,127]. Thus, loco-regional therapies for the treatment of colorectal liver metastases may promote CRC immunogenicity. This is probably also the result of the induction of tumor cell death and, as a consequence, increased tumor antigen presentation. As such, loco-regional therapies can have a positive effect on local and systemic anti-tumor immune responses.

## 7. Importance of CRC Immunogenicity for Novel Immune-Based Therapeutic Strategies

The new era of tumor immunology and immunotherapy can be of great additional value for the treatment of CRC. Cancer immunotherapy is aimed at stimulating the immune system through a variety of mechanisms to enhance anti-tumor immunity. It includes therapies directed to increase the activity and efficacy of effector immune cells such as T cells and NK cells, and to decrease activity of immunosuppressive Tregs, MDSCs, and TAMs. Current immunotherapeutic strategies include passive transfer of immune effectors such as targeted-monoclonal antibodies and T cells, or activation of endogenous tumor immunity by the use of cancer vaccination or modulation of immune checkpoints. Any successful immunotherapy requires an effective tumor-specific antigen presentation to effector T cells as well as additional co-signals regulating the level of T cell activation. Tumor antigens clearly participate in recognition and elimination of tumor cells by tumor-specific T cells in the context of immunotherapy [128]. The increasing knowledge of immunogenic tumor antigens in CRC offers important implications for the development of tumor-specific T cell immunotherapies.

In any immunotherapeutic approach, one should take into account and anticipate the above-mentioned tumor-immune system interactions. For instance, CRC patients that show low or absent MHC class I expression on their tumor cells are not expected to benefit from tumor-specific T cell stimulatory immunotherapies. Furthermore, the presence of immunosuppressive Tregs may hamper an effective tumor-specific effector T cell response. As such, CRC patients that show high levels of Tregs in the tumor microenvironment or circulation may benefit from the elimination of Tregs before administering cancer vaccines. Another option could be the use of selected sets of tumor antigens for cancer vaccines that induce optimal tumor-specific CD8^+^ T cell activity, but minimal tumor-specific Treg activity. Furthermore, the mutational load of CRC has been found to be highly predictive for the treatment response of immune-based therapies such as immune checkpoint inhibitors [112]. CRC patients exhibiting the MMR-deficient or MSI phenotype and, as a result, a high number of tumor-specific neo-antigens, were found to have clinical benefit from immune checkpoint inhibitor therapy [112].

To prevent selective outgrowth of CRC cells capable of escaping the immune system, combinational (immune-based) therapies may be highly valuable. The combination of blockade of immune inhibitory checkpoints with stimulation of immune-activating checkpoints such as OX40, CD27, or CD137 could result in the achievement of maximal activity of tumor-specific T cell responses [110]. Furthermore, cancer vaccines sequentially followed by immune checkpoint modulation can trigger pre-existing anti-tumor immune responses to tumor antigens by releasing tumor-associated inhibition of immune responses, thereby enhancing anti-tumor immunity in CRC patients [129]. Because of pro-immunogenic effects of conventional chemotherapy and radiation, there is a growing interest towards a novel role of chemotherapy and radiotherapy as “immunological adjuvants” [118]. In this context, chemotherapy and radiotherapy may potentially shift an immunosuppressive tumor microenvironment into an immunogenic one. The combination of chemotherapy or radiotherapy with modulation of immune checkpoints could thus be successful for the treatment of CRC. Finally, combinational therapies can be beneficial for the treatment of liver metastases of CRC patients. As discussed here, current loco-regional therapies for the treatment of liver metastases can result in tumor-specific local and systemic immune responses. Enhancement of their immune-stimulatory effects by combination with immunomodulatory antibodies targeting immune checkpoints may be effective.

Based on the present findings, the immune status of CRC is important for the efficacy of immunotherapies and may be modulated towards efficient anti-tumor immune responses. Since CRC immunogenicity varies between tumors and individuals, novel immune-based therapeutic strategies should not only anticipate the molecular profile, but also the immunological profile of a specific tumor.

## 8. Perspective

In this review, we discussed CRC immunogenicity in regards to tumor development and treatment. It is clear that CRC is more immunogenic than previously thought, and activates the immune system in various ways. The immunogenicity and activation of the immune system is, however, among other factors, dependent on expression of immunogenic tumor antigens, MHC molecules, ligands that bind to co-stimulatory or co-inhibitory receptors, tumor-specific T cells, mutational load of a tumor, and presence of immunosuppressive cells in the tumor microenvironment. As such, it is crucial to identify the interactions between a specific tumor and the host immune system in order to be able to direct the design of efficient immune-based therapeutic strategies. From an immunological perspective, tumor-immune system interactions differ in primary tumors, in the circulation, and in liver metastases. These different tumor-immune system interactions obviously affect tumor immunogenicity. Further analyses regarding the immunomodulatory interactions between tumor cells and immune cells are required, particularly regarding interactions in the circulation and liver metastases of CRC. Such studies may reveal novel strategies to overcome tumor escape and immunosuppression, which are currently major challenges for the development of efficient immune-based therapies for CRC. It is also important to study combinations involving different immunomodulatory therapies or combinations of immunomodulation with conventional chemotherapy and radiotherapy, which have been shown to promote CRC immunogenicity. New approaches that circumvent immunoediting of tumors may also be promising. Such an approach is the use of chimeric antigen receptor (CAR) T cells. One of the advantages of this approach is that no antigen presentation by HLA class I is required; the targeting antigen is a tumor cell surface protein. Although CAR T cells are mostly used in B cell malignancies, a few clinical trials in patients with solid tumors have been initiated [6,130]. Currently, anti-CEA, anti-HER2, and anti-MUC1 CAR T cells are studied in clinical trials. For anti-cancer therapies targeting CRC, understanding a specific type of tumor with its own unique molecular and immunological profile and its response to anti-tumor immune responses is essential.

## 9. Conclusions

The current knowledge on CRC identifies immunogenicity as a central issue and indicates the clinical significance of tumor-specific immune responses in CRC. However, the complexity of the multifaceted tumor-immune system interactions in primary tumors, in the circulation, and in liver metastases of CRC patients, as summarized in Figure 2, renders the translation to treatment for CRC difficult and demands further studies. For novel immune-based therapeutic strategies interfering in tumor-immune system interactions in CRC, it is obvious that one should take into account and anticipate not only the molecular profile, but also the immunological profile of a specific tumor.

## Abbreviations

CAR	Chimeric antigen receptor
CEA	Carcinoembryonic antigen
CIMP	CpG island methylator phenotype
CIN	Chromosomal instability
CRC	Colorectal cancer
CTLA-4	Cytotoxic T-lymphocyte-associated antigen 4
DC	Dendritic cell
EGFR	Epidermal growth factor receptor
EMT	Epithelial-mesenchymal transition
EpCAM	Epithelial cell adhesion molecule
HER2	Human epidermal growth factor receptor 2
HLA	Human leukocyte antigen
IHP	Isolated hepatic perfusion
MDSC	Myeloid-derived suppressor cell
MHC	Major histocompatibility complex
MMR	Mismatch repair
MSI	Microsatellite instability
MSS	Microsatellite stable
MUC1	Mucin 1
NK	Natural killer
NKT	Natural killer T
PD-1	Programmed death 1
PD-L1	Programmed death-ligand 1
PD-L2	Programmed death-ligand 2
sHLA	Soluble HLA
TAM	Tumor-associated macrophage
TIL	Tumor-infiltrating lymphocyte
TP53	Tumor protein 53
Treg	Regulatory T cell
